# Irvalec Inserts into the Plasma Membrane Causing Rapid Loss of Integrity and Necrotic Cell Death in Tumor Cells

**DOI:** 10.1371/journal.pone.0019042

**Published:** 2011-04-27

**Authors:** José M. Molina-Guijarro, Álvaro Macías, Carolina García, Eva Muñoz, Luis F. García-Fernández, Miren David, Lucía Núñez, Juan F. Martínez-Leal, Victoria Moneo, Carmen Cuevas, M. Pilar Lillo, Carlos Villalobos Jorge, Carmen Valenzuela, Carlos M. Galmarini

**Affiliations:** 1 Departamento de Biología Celular, PharmaMar S.A., Colmenar Viejo, Madrid, Spain; 2 Instituto de Investigaciones Biomédicas “Alberto Sols” (CSIC-UAM), Madrid, Spain; 3 Departamento Biofísica, Instituto de Química-Física “Rocasolano” (CSIC), Madrid, Spain; 4 Instituto de Biología y Genética Molecular (CSIC-UVA), Valladolid, Spain; Stanford University, United States of America

## Abstract

Irvalec is a marine-derived antitumor agent currently undergoing phase II clinical trials. In vitro, Irvalec induces a rapid loss of membrane integrity in tumor cells, accompanied of a significant Ca^2+^ influx, perturbations of membrane conductivity, severe swelling and the formation of giant membranous vesicles. All these effects are not observed in Irvalec-resistant cells, or are significantly delayed by pretreating the cells with Zn^2+^. Using fluorescent derivatives of Irvalec it was demonstrated that the compound rapidly interacts with the plasma membrane of tumor cells promoting lipid bilayer restructuration. Also, FRET experiments demonstrated that Irvalec molecules localize in the cell membrane close enough to each other as to suggest that the compound could self-organize, forming supramolecular structures that likely trigger cell death by necrosis through the disruption of membrane integrity.

## Introduction

Irvalec (Elisidepsin, PM02734) is a synthetic cyclodepsipeptide closely related to Kahalalide F, a natural antitumor compound isolated from the Hawaiian marine mollusc *Elysia rufescens* [1]. Preliminary in vitro and in vivo studies identified Irvalec as a new antiproliferative drug with activity against a broad spectrum of tumor types [Molina-Guijarro JM et al.; AACR Annual Meeting 2009; abstr 888]. In patients, the compound is well tolerated and does not show the signs of toxicity commonly observed with standard anticancer treatments [2]. Irvalec is currently in phase II clinical studies for squamous non-small cell lung cancer (NSCLC), gastric and esophageal cancer.

It was previously reported that Kahalalide F induces a rapid membrane permeabilization, with loss of mitochondrial membrane potential and of lysosomal integrity, and profound general alterations in the cells, including severe cytoplasmic swelling and vacuolization, dilatation and vesiculation of the endoplasmic reticulum, mitochondrial damage and plasma membrane rupture [3]–[5]. Other groups have reported that Kahalalide F interferes with different signaling pathways such as EGFR, HER2/neu, ErbB3, TGF-α or PI3K/AKT. Kahalalide F mediates a necrotic cell death type rather than apoptosis that is not associated with DNA degradation or cell cycle blockade [6].

Concerning Irvalec, a functional screening assay performed on a collection of 4,848 viable *Saccharomyces cerevisiae* haploid deletion mutants [7] showed that proteins involved in vesicle trafficking appear to be important for the activity of Irvalec. Thus, yeast cells defective in these pathways were more sensitive to the drug than their wild-type counterparts whereas a mutant strain lacking the sphingolipid fatty acyl 2-hydroxylase Scs7 (orthologue to human FA2H) was found to be the most resistant strain. In fact, overexpression of Scs7/FA2H in yeast or mammalian cells rendered them more sensitive to the drug. Although not yet fully understood, it seems that fatty acid 2-hydroxylation is important for the maintenance of membrane conformation and integrity in some tissues.

Here we show that the potent cytotoxic activity of Irvalec is exerted very rapidly through insertion of the drug molecule into the plasma membrane and induction of drastic loss of membrane integrity. As a result, severe cell swelling, formation of giant vesicles (GVs), a significant Ca^2+^ influx and alterations in cell membrane conductivity are detected. These membrane changes were not observed in tumor cells with acquired resistance to the compound. Moreover, these cytotoxic effects could be delayed by pretreating the cells with Zn^2+^, which has been described as a membrane protector [8]. These results indicate that Irvalec interacts directly with the cell membrane and induces a rapid and severe disorganization of the lipidic bilayer of tumor cells that disturbs the water-electrolyte balance causing necrosis.

## Materials and Methods

### Reagents

Irvalec (C77H125F3N14O18, MW:1591.89) (Figure S1) and its fluorescent derivatives were manufactured at PharmaMar, SA. Stock solutions (10 mM in DMSO) were prepared and stored at −20°C. Sulforhodamine B (SRB), 3-(4,5-dimethylthiazol-2-yl)-2,5-diphenyltetrazolium bromide (MTT), Trizma® base, Hoechst-33342, propidium iodide (PI), Dulbecco's modified Eagle's medium (DMEM), penicillin, streptomycin and fetal calf serum (FCS) were purchased from Sigma (St. Louis, MO, USA). Calcein acetoxymethylester (calcein-AM) was purchased from Calbiochem (Cambridge, MA, USA). AlexaFluor-488 conjugated β-subunit cholera toxin, and Fura2/AM were purchased from Invitrogen (Carlsbad, CA, USA). The lipophilic fluorescent probe 2-carboxyethyl-1,6-diphenyl-1,3,5-hexatriene (PA-DPH) was from Molecular Probes (Eugene, USA).

### Cell lines

A panel of cell lines was used representing the following cancerous tissue types: cervix (HeLa), prostate (PC-3 and 22Rv1), pancreas (PANC-1 and MiaPaca-2), ovary (IGROV-1, IGROV-1/ET and A2780), lung (NCI-H460, NCI-H23 and A549), liver (SK-HEP-1 and HepG2), leukemia, (MOLT-4 and K-562), renal (RXF 393 and Caki-1), gastric (HGC-27 and HS 746T), colon adenocarcinoma (LoVo, LoVo/Dox, HT-29 and HCT-116), and breast (MDA-MB-231, MCF-7 and BT-474). Human embryonic kidney cells (HEK-293) were cultured in DMEM (Gibco, Invitrogen) supplemented with 10% bovine fetal serum, penicillin-streptomycin (Sigma) and non-essential amino acids 1% as previously described (Arias et al., 2007). IGROV-1/ET and LoVo/Dox are cell lines that over-express the P-glycoprotein. All these cell lines were purchased from the American Type Culture Collection and grown with the appropriate culture media supplemented with 10% FCS, 1% penicillin and streptomycin and 2 mM L-glutamine.

The resistant variant A549-Irv was developed from the A549 lung cancer cells using stepwise increases of concentration of Irvalec over an 18-month period. The maximum concentration used during selection was 50 µM.

### Cell viability assays

Cells were seeded in 96-well microtiter plates and allowed to stand for 24 hours at 37°C and 5% CO_2_ in drug-free medium before treatment with vehicle alone or Irvalec at the concentrations and times indicated in the text. For cell survival quantification, MTT was added to the cell cultures for 8 h. Then, the culture medium was carefully removed and the colored formazan crystals dissolved in DMSO. The absorbance of the samples was measured at 540 nm using a microplate spectrophotometer. Results are expressed as percentage of control cell survival and represent the mean of at least three independent experiments. For short time treatment assays (30 min), a colorimetric assay using SRB, as previously described [9], was used.

### Membrane permeabilization assays

For time-course experiments, cells were seeded at high density in 96-well clear-bottom black plates and cultured as described above. When confluence was reached, fresh culture medium (supplemented with 25 mM HEPES pH 7.4 and 50 µg/mL PI) containing or not different concentrations of Irvalec was added. The uptake of PI was quantified by plate fluorimetry (531/632 nm) at 37°C, up to 70 min (1 min intervals), using a Victor-3 Multilabel Counter (Perkin Elmer). Results were expressed as relative fluorescent signals. In a different experimental setting, the cells were loaded with 1 µM calcein-AM for 15 min, then washed to remove excess calcein-AM and further cultured in medium containing 25 mM HEPES (pH 7.4) with or without Irvalec at the indicated concentrations. Calcein fluorescence decrease was monitored by fluorescence microscopy. Finally, cells were cultured in 60 mm Petri dishes and treated with vehicle alone or with the appropriate concentration of Irvalec in a final volume of 4 mL. For the quantification of the LDH (lactate dehydrogenase) enzymatic activity, the commercially available LDH-Cytotoxcity Assay Kit (BioVision) was used following the manufacturer's instructions.

### Cytosolic Ca^2+^ imaging

The effects of Irvalec on Ca^2+^ concentration dynamics were performed as previously described [10], [11]. Briefly, A549 (5×10^5^) cells were plated on 12 mm poly-L-Lysine-coated glass coverslips and loaded with 4 µM fura2/AM for 60 min at room temperature in 1 ml of standard medium containing 145 mM NaCl, 5 mM KCl, 1 mM CaCl_2_, 1 mM MgCl_2_, 10 mM glucose and 10 mM HEPES (pH: 7.42). Cells were then placed on the heated (37°C) stage of an inverted microscope (Zeiss Axiovert S100 TV). Cells were perfused continuously with 37°C pre-warmed standard medium and epi-illuminated alternately at 340 and 380 nm. Light emitted above 520 nm was recorded with an OrcaER digital camera (Hamamatsu Photonics, Shizuoka, Japan). Pixel by pixel ratios of consecutive frames were captured every 5 s.

### Electrophysiological recordings

The effects of Irvalec on membrane conductance were analyzed in A549, HCT-116, HEK-293 and HeLa cells as previously described [12]–[16]. Experiments were performed in a small bath mounted on the stage of an inverted microscope (Nikon model TMS, Garden City, NY) continuously perfused with the extracellular solution (Tyrode-glucose buffer). Ion currents were recorded at room temperature (20–22°C) using the whole-cell voltage-clamp configuration of the patch-clamp technique [17] with an Axopatch 1C patch-clamp amplifier (Axon Instruments, Foster City, CA). Currents were filtered at 2 kHz (four-pole Bessel filter), sampled at 4 kHz. Data acquisition and command potentials were controlled by the CLAMPEX program of PCLAMP 6.0.1 software (Axon Instruments). Micropipettes were pulled from borosilicate glass capillary tubes (Narishige, GD-1, Tokyo, Japan) on a programmable horizontal puller (Sutter Instrument Co., San Rafael, CA) and heat-polished with a microforge (Narishige). Pipette tip resistance averaged between 1 and 3 MΩ. The intracellular pipette solution contained (in mM): K-aspartate 80, KCl 50, phosphocreatine 3, KH_2_PO_4_ 10, MgATP 3, HEPES-K 10, EGTA 5 and was adjusted to pH 7.25 with KOH. The external solution contained (in mM): NaCl 130, KCl 4, CaCl_2_ 1.8, MgCl_2_ 1, HEPES-Na 10, and glucose 10, and was adjusted to pH 7.40 with NaOH. Measurements were performed using the CLAMPFIT program of PCLAMP 9.2.

### Two-photon fluorescence lifetime imaging

Two-photon fluorescence-lifetime imaging (2P-FLIM) of live cells was carried out on a MicroTime200 system (PicoQuant, Germany) coupled with an Olympus IX71 inverted microscope mounted with a 60× water-immersion objective NA1.2. Each pixel is represented by the intensity-weighted average fluorescence lifetime of the pixel total fluorescence intensity. Horizontal polarized excitation (Y direction in the X-Y microscope plane) was performed by a mode-locked, femtosecond-pulsed Ti:Sapphire laser (Mai-Tai, Spectra Physics,CA) operating at a repetition rate of 80 MHz and tuned to 755 nm. For vertical polarization imaging the excitation polarization was interchanged from horizontal to vertically using a half-wave plate. Two-color (Oregon Green 488 and AlexaFluor 555) fluorescence images were acquired simultaneously with two single-photon avalanche diodes (SPAD, SPCM-AQR-14, Perkin Elmer, USA), through a dichroic beam splitter FF560-Di01 and bandpass filters FF01-520/35, FF01-607/36 or 685/40 (Semrock, Germany). Horizontal and vertically polarized fluorescence images were acquired simultaneously using a polarization beamsplitter cube. The excitation power (0.8–8 mW before the objective) was adjusted using a variable optical attenuator LS-107AT (Lasing, S.A. Spain) to achieve rates of counting lower than 10^6^ photons/s. FLIM X-Y scans were recorded at different Z values with a piezo XY-scanning table and PiFoc Z-piezo (E-710 Digital PZT controller, PI, Germany), with the time-correlated single-photon counting (TCSPC) technique, by using a TimeHarp 300 PC-board (PicoQuant, Germany), synchronized with the laser pulses. Acquisition time per pixel accounted for 0.6 ms–1.2 ms, resulting in image overall acquisition time of 60 s–180 s, depending on the image resolution and the intensity of the fluorescence signal.

A549 cells grown on LabTek chambered coverglass slides (Thermo Scientific-Nunc) were washed and incubated with Tyrode-glucose buffer containing PA-DPH (0.5 µM) at 22°C for 10 min in the dark. In the assays performed with Irvalec-Oregon Green 488 (Irv-OG488) and Irvalec-AlexaFluor 555 (Irv-A555) stock solutions were 0.5 mM (in 100% DMSO) and were added to the Tyrode-glucose buffer to the appropriate final concentration, keeping DMSO at 0.4% v/v.

### Statistical methods

Results are expressed as mean ± SEM. Direct comparisons between mean values in control conditions and in the presence of drug for a single variable were performed by paired Student's t-test. Student's t-test was also used to compare two regression lines. Differences were considered significant if P<0.05.

## Results

### Cytotoxicity of Irvalec against human tumor cells

The cytotoxicity of Irvalec was studied in vitro in a panel of 23 human tumor cell lines, derived from 11 different tissues. Dose-response curves were performed at 72 h and cell survival quantified using the MTT method. Irvalec displayed cytotoxic activity with a mean IC_50_ value of 2.3 µM. Table 1 shows the relative IC_50_ values obtained for each cell line. Prostate (PC3 and 22RV1) and pancreas (PANC-1 and MIA-PaCa) cell lines tended to be the most and least sensitive, respectively. Cell lines over-expressing the P-glycoprotein (IGROV-1/ET and LoVo/Dox) presented similar IC_50_ values than their parental counterparts (IGROV-1 and LoVo) indicating that the compound is not a substrate of this efflux pump.

**Table 1:** Cytotoxicity of Irvalec in a panel of 25 human cancer cell lines.

	Cell line*	IC_50_ (µM)
**Prostate**	PC3	0.6±0.3
	22RV1	0.3±0.2
**Pancreas**	PANC-1	>6.3±0
	MiaPaCa-2	>6.3±0
**Ovary**	IGROV-1	1.1±0.4
	IGROV-1/ET*	0.9±0.1
	A2780	1.3±0.2
**Lung**	NCI-H460	5.5±0.3
	NCI-H23	1.5±1.4
	A549	1.8±0.5
**Liver**	SK-HEP-1	1.5±0.7
	HEPG2	0.3±0.1
**Leukemia**	MOLT4	5.6±0.4
	K562	2.9±0.1
**Kidney**	RXF393	2.0±1.5
	CAKI-1	2.4±0.8
**Stomach**	HS746T	1.5±0.1
	HGC-27	1.3±0.6
**Colon**	LoVo	0.3±0.1
	LoVo/Dox*	0.5±0.1
	HT29	0.4±0.1
	HCT-116	3.6±1.8
**Breast**	MDA-MB-231	4.3±1.1
	MCF-7	0.9±0.2
	BT-474	0.3±0.1

*All cell lines were treated with increasing concentrations of Irvalec and cell growth inhibitory concentration (IC_50_) was determined after 72 h by MTT method. Values represent mean ± SD of three different experiments.

**Cell lines over-expressing the P-glycoprotein.

### Irvalec induces a necrotic cell death by affecting the cell membrane

**Figure 1:**
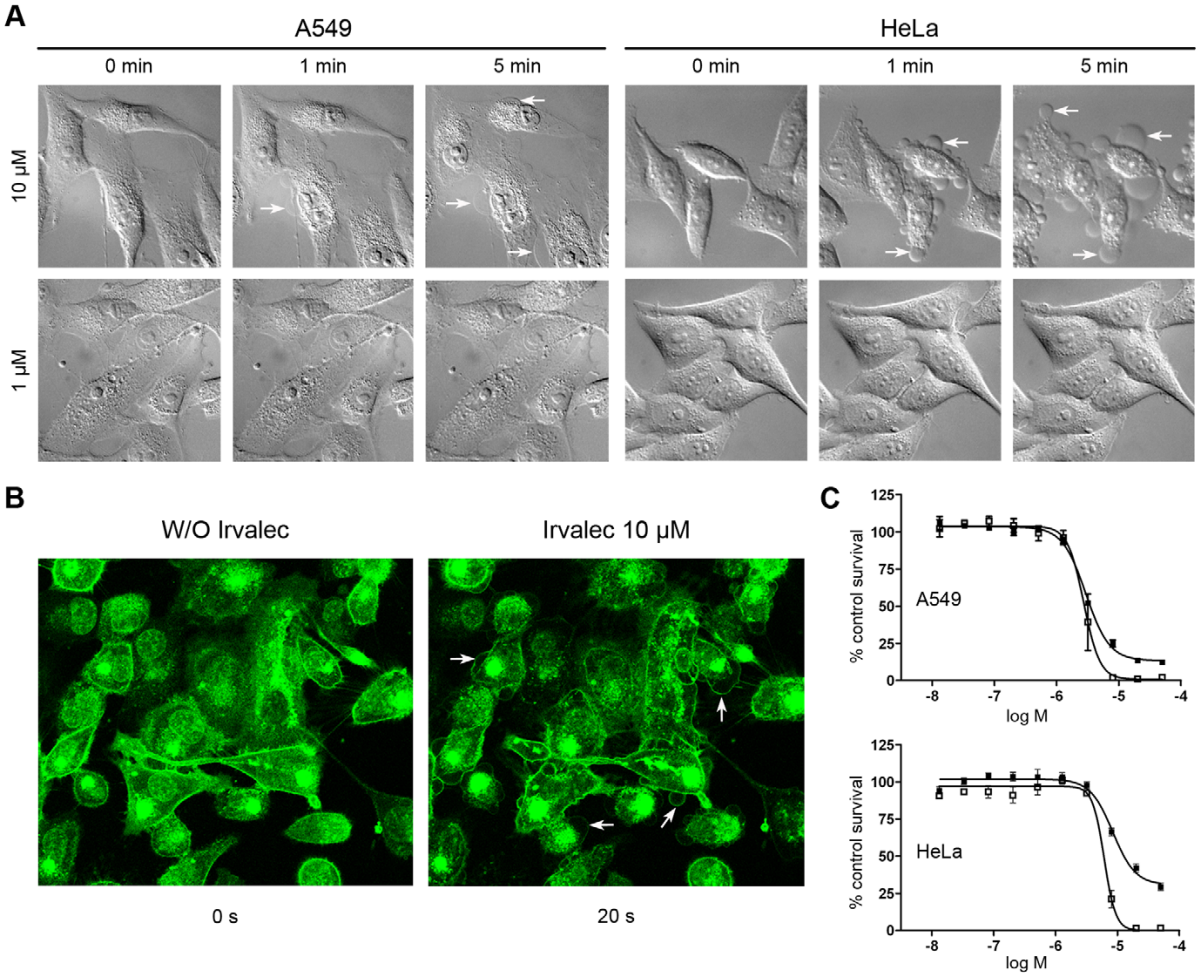
Irvalec induces a necrotic cell death. (A) Representative images of A549 and HeLa cells treated with Irvalec (1 and 10 µM) and examined by phase contrast video-microscopy; pictures were taken 1 and 5 min after treatment. (B) Effects of 10 µM Irvalec at the cell membrane A549 cells pre-treated with AlexaFluor 488-conjugated β-subunit of the cholera toxin to fluorescently label the plasma membrane; white arrows indicate the formation of giant vesicles (C) Dose-response cytotoxicity curves to analyze the activity of Irvalec after 30 min (▪) and 72 h (□) incubation times using the SRB method. Results represent the mean±SD of at least three different experiments.

A549 (lung) and HeLa (cervix) cells were treated with Irvalec (1 and 10 µM) and examined by phase contrast microscopy. While at 1 µM there was any visible effect, at 10 µM both cell lines showed similar rapid morphological changes. Within a few minutes, treated cells showed evident membrane destabilization as revealed by the appearance of cell swelling and abundant membrane blebs and GVs (Figure 1A and Video S1). These morphological changes were associated with necrotic cell death.

We then confirmed microscopically the effect of Irvalec on the cell membrane by pre-treating A549 cells with fluorescent β-subunit of the cholera toxin to label the plasma membrane, and then treating them with 10 µM Irvalec. As shown in Figure 1B and Video S2, cell GVs were clearly surrounded by fluorescently-labelled plasma membrane. These effects were plasma membrane-specific, since other cellular structures, such as lysosomes, were not affected by the compound at short treatment times (Figure S2). We finally performed parallel concentration-response cytotoxicity curves at two different drug exposure times, 30 minutes and 72 hours, and observed a perfect overlaping of the curves (IC_50_ values of 3.5 µM and 1.8 µM, respectively) (Figure 1C), indicating that the cytotoxic activity of Irvalec was directly associated with its early “all” or “non” effects on the cell membrane.

### Irvalec induces rapid membrane permeabilization in tumor cells

**Figure 2:**
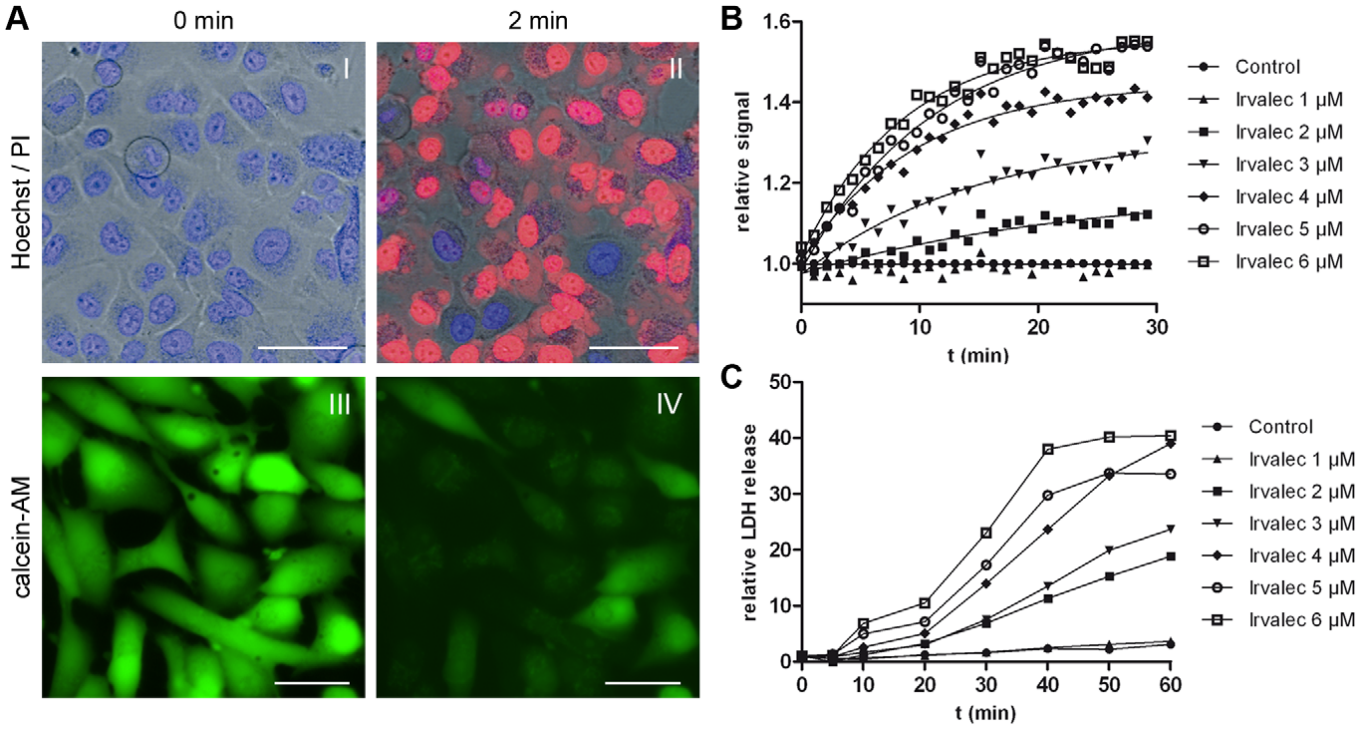
Irvalec induces a concentration-dependent rapid membrane permeabilization. (A) Representative images of A549 cells exposed to Irvalec 10 µM for 2 min. In the presence of propidium iodide (PI), unpermeabilized cells show intact nuclei (I) while permeabilized cells show PI stained nuclei (II); in pre-loaded with calcein AM cells, the intracellular fluorescence (III) rapidly vanished from permeabilized cells upon Irvalec treatment (IV) (scale bar: 50 µm) (B) Time-course of Irvalec-dependent membrane permeabilization of A549 cells using different concentrations of the drug (ranging from 1 to 6 µM) as assessed by plate fluorimetry using PI nuclear staining; (C) Time-course of Irvalec-dependent membrane permeabilization of A549 cells using different concentrations of the drug (ranging from 1 to 6 µM) as assessed LDH release.

We then evaluated whether exposure to Irvalec was associated with a rapid loss of cell membrane integrity. A549 cells were cultured in the presence of PI and exposed to the drug for different time intervals. As shown in Figure 2A and Video S3, a rapid PI uptake occurred after 2 minutes treatment with 10 µM Irvalec. To confirm these results, A549 cells were pre-loaded with calcein-AM, a permeable derivative of fluorescein. While non-treated cells remained fluorescent, Irvalec treatment induced a rapid loss (2 min) of fluorescence (Figure 2A; Video S4). Again, cell membrane permeabilization was not observed at low concentrations even at long treatment periods (>24 h) (data not shown).

We also studied the kinetics of membrane permeabilization mediated by Irvalec in time-course experiments. A549 cells were cultured in the presence of PI and exposed or not to different concentrations of Irvalec (from 1 to 6 µM). The nuclear PI fluorescence was measured in real time (1 min intervals) by fluorometry. As shown in Figure 2B, Irvalec affected the permeability of tumor cells in a time- and concentration-dependent manner. At concentrations of 2–3 µM, Irvalec had low to moderate permeabilization effects that showed a linear dependency with time. At higher concentrations, 4–6 µM, the effect of the drug was more drastic insofar as a significant loss of membrane permeability was observed within the first minutes after treatment. We finally analyzed the release of intracellular LDH into the extracellular culture media as a measure of cell membrane permeabilization. Cells were treated with Irvalec (1–6 µM) and aliquots of the supernatants were taken at different time intervals to evaluate the levels of LDH. As shown in Figure 2C, Irvalec induced the release of LDH in a time- and concentration-dependent manner, with a slower kinetics than that observed for PI. Altogether, these results demonstrated that Irvalec induces rapid membrane damage that alters the selective permeability of cells to molecules of very different sizes (PI, calcein or LDH).

### Irvalec elicits free Ca^2+^ movements across the cell membrane

**Figure 3:**
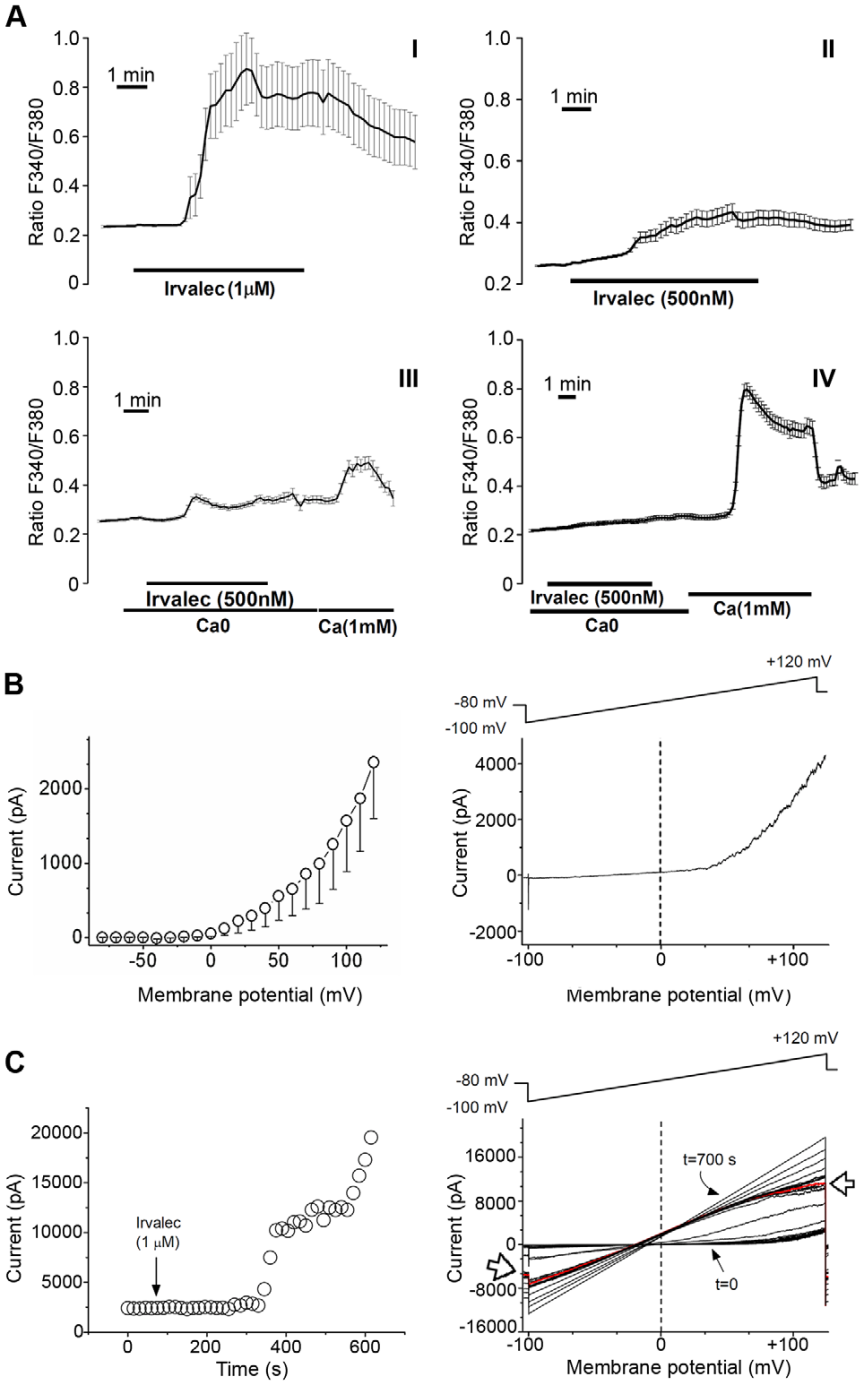
Effects of Irvalec on cytosolic Ca^2+^ concentrations and cell membrane conductivity in A549 cancer cells. (A) Representation of cytosolic Ca^2+^ concentration imaging records (F340/F380 ratio) in A549 cells. (I) Alteration in cytosolic Ca^2+^ after treatment with Irvalec 1 µM (n = 12); the panel corresponds to the mean ± SEM values of [Ca^2+^]cyt in all cells for each experiment each microscopic field (II) Representation of cytoplasmic Ca^2+^ concentration records in A549 cells after treatment with Irvalec 0.5 µM (n = 29); the panel corresponds to the mean ± SEM values of [Ca^2+^]cyt in all cells for each experiment (III) Effects of removal of extracellular Ca^2+^ on the rise in cytosolic Ca^2+^ induced by 0.5 µM Irvalec; the graph corresponds to the mean ± SEM values of [Ca^2+^]cyt in all cells (n = 31) for two different experiments (IV) Effects of depletion of intracellular Ca^2+^ stores with 1 µM thapsigargin during 10 min on the rise in cytosolic Ca^2+^ induced by 500 nM Irvalec in Ca^2+^-free medium and after re-addition of extracellular calcium; the panel corresponds to the mean ± SEM values of [Ca^2+^]cyt in all treated cells (n = 29). (B) Electrophysiological characteristics of A549 cells; left panel shows the current-voltage relationship obtained after applying the pulse protocol described in Experimental Procedures. Amplitude of the potassium currents were measured at the end of 250 ms depolarizing pulses and were represented versus membrane potential. Right panel shows the ion current elicited after applying a ramp pulse protocol from −100 mV to +120 mV during 500 ms from a holding potential of −80 mV (C) Electrophysiological effects of 1 µM Irvalec; left panels shows the amplitude of the maximum current at the end of the ramp. Note that the downward of the current observed in the left panel is reflected in a plateau phase in the increase of the current; right panel shows original records after applying a ramp pulse protocol from −100 mV to +120 mV during 500 ms; white arrows shows a downward in the current.

Next, we investigated the effects of 0.5 and 1 µM Irvalec on cytosolic Ca^2+^ concentrations ([Ca^2+^]cyt) in Fura2-loaded A549 cells. As shown in Figures 3A-I and 3A-II, Irvalec treatment promoted a rapid and large, concentration-dependent rise in ([Ca^2+^]cyt) at both concentrations. This was evidenced as shown by the increase in the Fura2 fluorescence emission when samples were excited at 340 nm and the decrease in Fura2 fluorescence emission when the sample was excited at 380 nm leading to a large rise in the ratio of both fluorescences (F340/F380 ratio). Noteworthy, after treatment of A549 cells with both Irvalec concentrations, some cells presented a sudden decrease of the F340/F380 ratio probably caused by the cell permeabilization and loss of the dye. To further investigate the permeability to Ca^2+^ in A549 cells we used Mn^2+^ as a surrogate for Ca^2+^. Its permeability can be followed in fura2 loaded cells because Mn^2+^ quenches fura2 fluorescence emissions at all excitation wavelengths. Treatment with 0.5 µM Irvalec quickly promoted sudden decreases in the F340/F380 ratio in the presence of Mn^2+^ in A549 cells (Figure S3).

We then investigated whether the rise in [Ca^2+^]cyt was due to extracellular or intracellular sources. For this purpose, A549 cells were treated with 0.5 µM Irvalec in the absence of extracellular Ca^2+^. Figure 3A-III shows that, under these conditions, Irvalec was able to elicit only a small and transient rise in [Ca^2+^]cyt. Re-addition of extracellular Ca^2+^ resumed the larger, typical effect of Irvalec, suggesting that the rise in [Ca^2+^]cyt was mainly due to extracellular Ca^2+^. When depleting intracellular Ca^2+^ stores using thapsigargin (1 µM for 10 min) in Ca^2+^-free medium, 0.5 µM Irvalec induced almost no change in [Ca^2+^]cyt thus confirming that the Ca^2+^ was mainly coming from extracellular sources (Figure 3A-IV). Again, re-addition of extracellular Ca^2+^ promoted a every large rise in cytosolic Ca^2+^.

### Irvalec disturbs cell membrane conductivity

As membrane damaging agents act by disrupting the normal functioning of cell membranes, we also analyzed the effect of the non-cytotoxic concentration of 1 µM Irvalec on membrane conductivity in A549 cells. Figure 3B shows the current-voltage relationship (IV) of the endogenous outward K^+^ current of A549 cells recorded after applying depolarizing 250-ms pulses from a holding potential of −80 mV. The magnitude of this current exhibited a mean value of 1080±355 pA (n = 8), when cells were pulsed from a holding potential of −80 mV to +60 mV and measured at the end of a 250-ms pulse. The same figure also shows (right panel) the current elicited after applying a ramp protocol from −100 mV to +120 mV during 500 ms [18].

In order to analyze the effects of 1 µM Irvalec, a series of ramps from −100 mV to +120 mV during 500 ms were applied. As shown in Figure 3C, Irvalec produced an increase of the membrane conductance at all membrane potentials that led to the appearance of an in- and an out-ward current at negative and positive membrane potentials, respectively. During the appearance of Irvalec effects, a downward effect of the current during the application of the ramp protocol was also observed (white arrows). This type of effect is also observed when Kv1.5 channels are co-expressed with the Kvβ1.3 subunit that induces a fast and incomplete inactivation of the current [14], [19]–[21]. Also as shown in Figure 3C, the time effects of Irvalec appeared within two phases, due to the observed downward effect. The time lag between the application of Irvalec and the beginning of the effects was 440±45 s (n = 4). Similar effects were observed when exposing HEK-293, HeLa and HCT-116 cells to 1 µM Irvalec (data not shown).

### Zinc salts protect cells against the early cytotoxic effects of Irvalec

**Figure 4:**
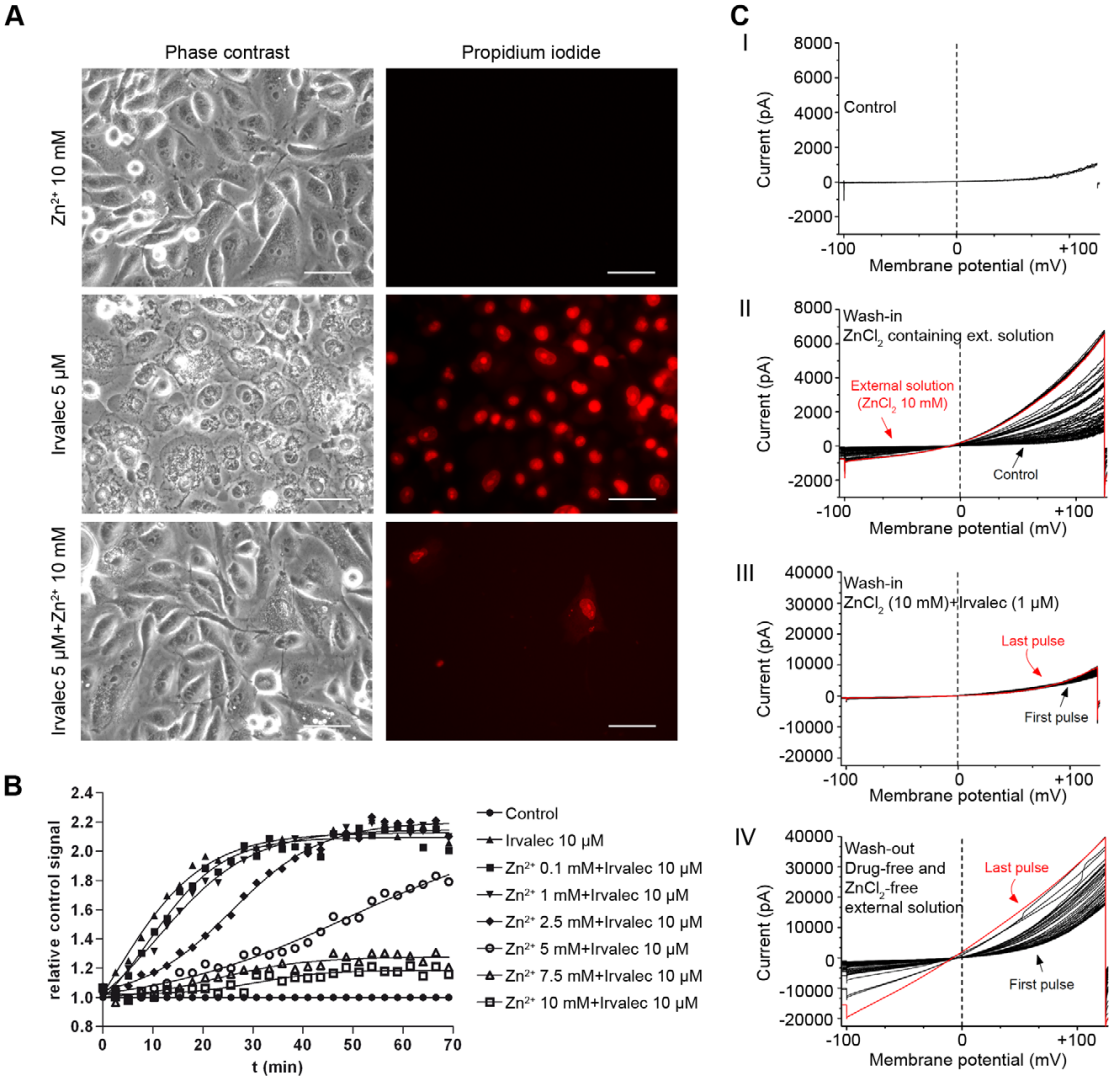
Zinc protects tumor cells against Irvalec cytotoxicity. (A) Representative images of A549 cells treated with 5 µM Irvalec alone or in combination with 10 mM ZnCl_2_, in the presence of PI. Cell permeabilization was followed by fluorescence microscopy (red nuclear staining) (scale bar: 50 µm) (B) Quantification of Irvalec induced cell permeabilization in real time, using plate fluorimetry. A549 cells were treated with 10 µM Irvalec alone or in combination with different concentrations of ZnCl_2_ (ranging from 0.1 mM to 10 mM) in the presence of PI. Nuclear staining was measured at intervals of 1 min and represented as relative signal from control fluorescence (C) Effect of ZnCl_2_ on the electrophysiological recording obtained in A549 cells treated with Irvalec; cells were treated with the following solutions in a sequence, I: control conditions, II: ZnCl_2_ (10 mM), III: 1 µM Irvalec in the presence of 10 mM ZnCl_2_ and IV: ZnCl_2_-free and Irvalec-free external solution.

Since Zn^2+^ has well established membrane-stabilizing properties, we investigated whether this cation could protect tumor cells from Irvalec cytotoxicity. A549 cells were pre-treated or not with 10 mM ZnCl_2_ (a non-toxic concentration) and then treated with 5 µM Irvalec for 15 min in the presence of PI. As shown in Figure 4A, treatment with Irvalec alone resulted in a rapid and massive cell death. In contrast, cells pretreated with ZnCl_2_ were significantly protected (Figure 4A and Video S5). In the absence of ZnCl_2_, approximately 60% of the cells were permeabilized by Irvalec while in its presence, less than 5% were affected. This represented a protection against Irvalec cytotoxicity in more than 90% of the cells that otherwise would be affected by the drug.

The ZnCl_2_ protective effect against Irvalec was also quantified by means of PI fluorimetry experiments (Figure 4B). A549 cells were treated with 10 µM Irvalec alone or in combination with different concentrations of ZnCl_2_ for 70 minutes. PI staining was analyzed by real-time plate fluorimetry (1 min intervals). ZnCl_2_ had a dose-dependent protective effect. At concentrations below 2.5 mM, ZnCl_2_ had a minor effect, exerting low or no protection at all. At 5 mM, a partial protective effect was observed, while at higher concentrations, 7.5 or 10 mM, the protection exerted by ZnCl_2_ was nearly complete during the duration of the experiment. Similar results were obtained when ZnSO_4_ was used in lieu of ZnCl_2_ (data not shown).

We finally perfused A549 cells with several solutions and measured the cell membrane conductivity in the following order: control (Figure 4C-I), external solution containing 10 mM ZnCl_2_ (Figure 4C-II), external solution containing 10 mM ZnCl_2_ and 1 µM Irvalec (Figure 4C-III) and control external solution again (Figure 4C-IV). Figure 4C shows the results of a typical membrane conductivity experiment under these conditions (n = 3). Treatment with 10 mM ZnCl_2_ produced changes both in the magnitude and gating of the endogenous Kv1.5 channel expressed by these cells (Figure 4C-II). As shown in this Figure, ZnCl_2_ increased the magnitude of the current when measured at +120 mV (at the end of the ramp) and shifted the threshold of the activation of the current to more negative potentials (data not shown). After washing the A549 cells with an external solution containing ZnCl_2_ and Irvalec (Figure 4C-III) a slight increase in the outward current was observed without any increase in the inward conductance of the current observed in the absence of ZnCl_2_ (Figure 3B). Only during the wash-out with a ZnCl_2_- and Irvalec-free external solution, the typical effects of Irvalec were observed (Figure 4C-IV). Similar results were obtained in HCT-116 and HEK-293 cell lines (data not shown). Altogether, these experiments clearly indicated that strengthening the cell membrane architecture with zinc salts diminished the drastic effects of Irvalec on the cell membrane.

### Characterisation of A549 cells resistant to Irvalec

**Figure 5:**
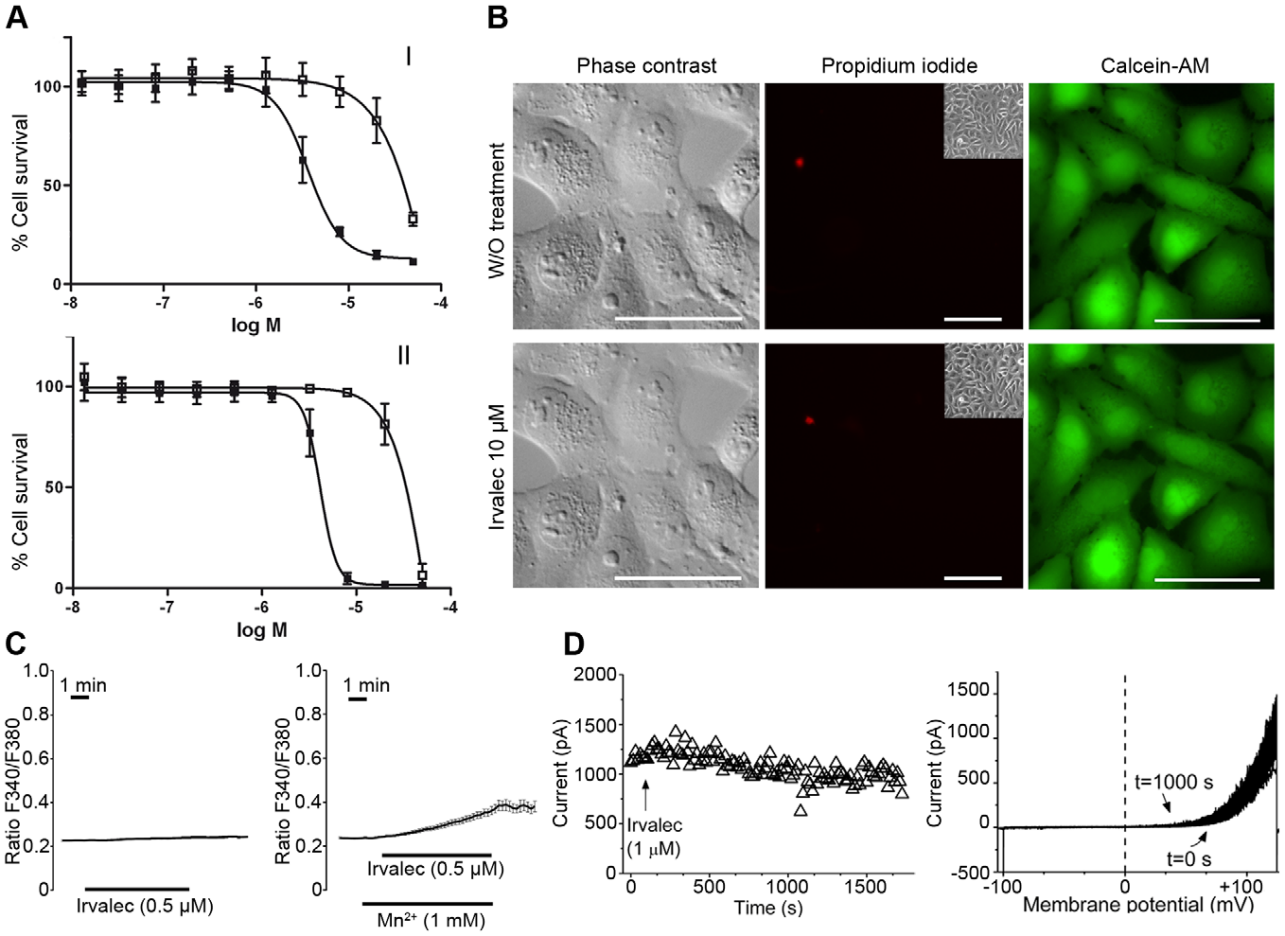
Characterization of Irvalec-resistant A549-Irv cells. (A) Dose-response cytotoxicity curves to analyze the activity of Irvalec in A549-Irv after exposure of 30 minutes (I) and 72 h (II); results represent the mean±SD of at least three different experiments (B) Effects of Irvalec treatment in cell membrane permeabilization; representative images of A549-Irv cells exposed to vehicle or to 10 µM Irvalec for 10 min are shown. Resistant cells kept cell morphology, did not show propidium iodide staining and retained preloaded calcein-AM in the cytoplasm (scale bar: 50 µm) (C) Effects of Irvalec treatment on cytosolic Ca^2+^; the left panel shows the effects of 0.5 µM Irvalec on cytosolic Ca^2+^ concentration (n = 27); the right panel represents the fura2 F340/F380 ratio in the presence of Mn^2+^ after treatment with 0.5 µM Irvalec (n = 16) (D) Effects of Irvalec treatment on cell membrane conductivity; left panel shows original records after applying a ramp pulse protocol from −100 mV to +120 mV during 500 ms. Right panel shows the amplitude of the maximum current at the end of the ramp.

A549-Irv cells were originated from A549 parental cells by a classical stepwise selection procedure. As shown in Figure 5A, cells were more than 30-fold more resistant to Irvalec than the parental A549 cells. The IC_50_ values after 30 min or 72 h exposure were 55±0.5 µM and 34±0.5 µM, respectively (Figure 5A-I and 5A-II). This cell line did not express efflux pumps on the cell membrane and did not show any cross resistance with other common anticancer agents (Figure S4 and Table S1). Incubation of A549-Irv with 10 µM Irvalec did not alter the cell membrane integrity and did not induce necrotic cell death (Figure 5B) as occurred in the parental A549 cells (Figures 1 and 2). Irvalec treatment also failed to induce any rise in [Ca^2+^]_cyt_ in the resistant subline at concentrations of 0.5 µM or 1 µM (Figure 5C). Addition of Mn^2+^ 1 mM did not induce any decreases in the F340/F380 ratio, as expected (Figure 5C). As shown in the current records, A549-Irv cells were completely insensitive to 1 µM Irvalec effects, as no changes in membrane conductance were observed (Figure 5D). Altogether, these results indicated that in A549-Irv cells Irvalec treatment was unable to induce any alteration in cell membrane integrity.

### Irvalec inserts into the plasma membrane

Since the effect of Irvalec was associated with rapid and dramatic changes in the plasma membrane integrity, we used two-photon time-resolved (TFTR) imaging to investigate whether the compound directly integrated into the plasma membrane. First, we took advantage of the characteristics of the lipophilic dye PA-DPH to obtain information about the effect of Irvalec on the lipid reorganization of the plasma membrane. PA-DPH localizes to the plasma membrane, placed parallel to the lipid acyl chain axis, with average fluorescence lifetimes of about 6.6 ns, and no partition preference between the gel and fluid phase phospholipids is shown. Interestingly, this cylindrical molecule has absorption and fluorescence emission dipoles aligned parallel to its long molecular axis that allows measurements of lipid order when polarized light microscopy is used.

**Figure 6:**
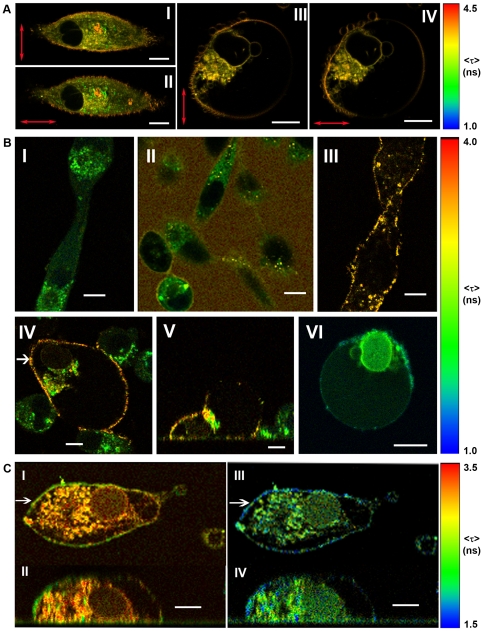
Interactions between Irvalec and the plasma cell membrane. For these experiments, A549 cells were grown on LabTek chambered slides; 2P-FLIM images are representative of different experiments. (A) Visualization of changes in the plasma cell membrane organization in A549 cells without (I and II) or after treatment (III and IV) with 2 µM Irvalec. A549 cells were labelled with 1 µM PA-DPH and excited with polarized light microscopy. The direction of the linear polarized excitation beam is shown in the figure with red arrows: Horizontal (I and III) and Vertical (II and IV); Emission filter: 483/32; I–II: (XY section, Z = 0), III–IV (XY section, Z = 5 µm) (B) Localization of Irvalec in the cell membrane; A549 cells were treated with Irv-OG488 or Irv-A555 and analyzed by two-photon time-resolved fluorescence microscopy; I: untreated cells (XY section, Z = 0); II: cells treated with 0.6 µM of Irv-OG488 (XY section, Z = 0); III: cells treated with 2.2 µM of a mix of Irv-OG488 and Irvalec (1∶4.5) (XY image, Z = 0); IV and V: cells treated with 0.4 µM of a mix of Irv-OG488 and Irvalec (1∶3) (IV: XY image, Z = 5 µm; V: XZ section, Y position defined by white arrow in IV); VI: cells treated with 2 µM of a mix of Irv-A555 and Irvalec (1∶4). (XY section, Z = 10 µm); emission bandpass filters; I–V: FF01-520/35; VI: FF01-607/36; Total intensity scale in arbitrary units: 0–1(I, IV–VI); 0–2(II); 0–50 (III). (C) Representative example of time-resolved fluorescence resonance energy-transfer (FLIM-FRET) experiments in A549 cell exposed to a mix of 0.6 µM Irv-OG488, 1.8 µM Irv-A555 and 2.4 µM Irvalec; I (XY section, Z = 0) and II (YZ section, X position defined by white arrow in I): donor channel image (OG488); III (XY section, Z = 0) and IV (YZ section, X position defined by white arrow in III): acceptor channel image (A555); donor filter: FF01 520/35; acceptor filter: FF01 685/40; dichroic beam splitter: FF560-Di01; total intensity scale in arbitrary units: 0–7 (I–II); 0–1 (III–IV). Scale bar: 10 µm.

Adherent cultures of A549 cells were grown on chambered slides, washed and labeled with 1 µM PA-DPH, treated with 2 µM Irvalec, and observed with TFTR fluorescence microscopy. Figure 6A shows representative 2P-FLIM pictures of the experiment. PA-DPH emission is distributed near homogeneously all over the plasma membrane of A549 untreated cells with intensity average fluorescence lifetimes of about 4.5 ns. The acquired 2P-FLIM images, both in the horizontal (Figure 6A-I) and vertical (Figure 6A-II) polarization excitation directions, show details of the roughness of the plasma membrane of untreated cells at sub-µm resolution. These polarization images also show that the cell membranes kept a fluidic phase, in which the PA-DPH molecules still have freedom of movement. Furthermore, PA-DPH labels internal membranes/vesicles or cytoplasm components with an average fluorescence lifetime of about 3 ns (green-yellow colors in Figures 6A-I and -II). A549 cells treated with Irvalec showed membrane restructuring, and displayed GVs in which the dye lost freedom of movement and was efficiently excited only when the light polarization was perpendicular to the bilayer plane (Figures 6A-III and -IV). The GVs in Irvalec-treated cells were clearly labeled by PA-DPH and showed the strongest fluorescence in the direction parallel to the excitation polarization. Since this effect is directly related with the organization of the phospholipids, these results indicate that the lipids in the GVs are highly ordered. In the fluid phase, the photoselection effect would decrease because of the relatively low lipid order. Thus, Irvalec affected the fluidity of the plasma membrane phospholipids, inducing the formation of GVs in which the ordered phase predominates over the fluid phase.

We then used a fluorescently labeled Irvalec analog (Irvalec-Oregon Green 488; Irv-OG488) (Figure S5A) to further investigate the membrane effects of the drug. Adherent cultures of A549 cells were grown on chambered slides before being treated with Irvalec (Figure 6B-I). Then, cells were washed and treated with Irv-OG488 in Tyrode-glucose buffer and analyzed by fluorescence microscopy (Figure 6B-II to -VI). Surprisingly, when observed with TFTR microscopy, most of the A549 cells treated with non-cytotoxic concentrations of Irv-OG488 did not show any significant fluorescence in the plasma membrane (Figure 6B-II). Only a residual minor fluorescent labeling was observed in the extracellular fluid. The lack of fluorescence in the plasma membranes could be due to self-quenching of the dye when the Irv-OG488 molecules are (somehow) packed. After addition of unlabeled Irvalec, at cytotoxic concentrations, the fluorescence signal rapidly appeared in the plasma membrane (Figure 6B-III), probably due to some kind of organization of membrane-bound Irvalec molecules in the lipid bilayer. Then, all of a sudden, GVs started to form and cells died in a necrotic process (Figures 6B-IV and -V). The plasma membrane of affected cells only appeared labeled when a mix of labeled and unlabeled Irvalec was added. This was taken as an indication that Irv-OG488 may be organized in multimeric complexes in the affected plasma membrane. When cells were treated with a mix of Irv-OG488 and unlabeled Irvalec, the latter presumably intercalate between the former, thus avoiding OG-488 self-quenching of Irv-OG488 multimers. The same effect was observed when cells were treated with the AlexaFluor 555 derivative of Irvalec, Irv-A555 (Figure 6B-VI). In contrast, A549-Irv cells treated with a mix of Irv-OG488 and unlabeled Irvalec (final concentration: 4.6 µM) did not show any significant fluorescence in the plasma membrane; all the fluorescence came from the media and cells images appear as negative images (Figure S6A). Moreover, treated A549-Irv cells did not present any severe swelling or formation of giant membranous vesicles. Of note, a careful screening of the whole preparation allowed us to find a low percentage of cells (<5%) that showed Irv-OG488 interacting with the plasma membrane (Figure S6B); this specific cell population showed a much lower fluorescence signal of Irv-OG488 compared with the A549 sensitive cells in the same experimental conditions. Taken together, these results suggest that, at non-cytotoxic concentrations, Irvalec is evenly distributed throughout the cell membrane but forms some kind of assembly after a critical concentration is reached in A549 sensitive but not in the A549-Irv cells.

To further investigate whether Irvalec was evenly distributed through the cell membrane or was forming patches within the lipid bilayer at different drug concentrations, A549 adherent cells were treated with 0.5 µM Irv-OG488 (energy donor), 1.8 µM Irv-A555 (energy acceptor; Figure S5B) and 2.4 µM Irvalec. We then performed time-resolved fluorescence resonance energy transfer (FLIM-FRET) experiments. As shown in Figure 6C, both fluorescent Irvalec derivatives localized mainly to the plasma membrane, although some amount of intracellular fluorescence was also observed. The intensity-average fluorescence lifetime of Irv-OG488 bound to the plasma membrane shifts to the green (donor channel, Figure 6C-I and -II) due to resonant energy transfer to Irv-A555 molecules in the very near vicinity (distances OG488-A555 lower than 50 Å).

Importantly, both fluorescent compounds form patches in the plasma membrane, and in some specific intracellular locations, near the nucleus, where the chromophores were close enough (around 50 Å) to efficiently permit FRET. This effect was not seen in the intracellular membranes stained with both dyes, probably due to more unstructured insertions of the compounds that remained at longer distances. Also, we did not observe FRET when cells were treated with a mix of Irv-OG488 and Irv-A555 at non-cytotoxic Irvalec concentrations (data not shown), which confirms that Irvalec molecules were only grouped in the plasma membrane at cytotoxic concentrations.

## Discussion

Here we have shown that low micromolar concentrations of Irvalec induced a rapid cell death that occurs within minutes after drug treatment and in a wide variety of cell types. The characteristic “z” shape of the dose-response curves usually obtained with Irvalec, suggested that the drug needs to achieve a critical concentration in the cell to exert its cytotoxic action. Through dose-response cytotoxicity curves, we demonstrated that the short- (30 min) and long-term (72 h) effects of Irvalec on cell viability were virtually the same. This observation can be taken as an indication that the compound induced a drastic disruption of cellular homeostasis within minutes after treatment. Moreover, the fact that other organelles such as lysosomes were apparently not affected at short time exposures, suggests that Irvalec is acting primarily on the plasma membrane.

The rapid mode of cell killing after cell membrane damage is normally associated with a necrotic cell death, which is characterized by cellular swelling, plasma membrane rupture and loss of intracellular contents. All of these morphological and physiological events were confirmed in cells treated with Irvalec. Shortly after treatment with effective concentrations of the drug, a visible swelling process began, accompanied by massive blebbing and complete loss of membrane integrity, as evidenced by the permeabilization to PI. Loss of membrane integrity was further evidenced by the detection of intracellular components, such as LDH or pre-loaded calcein in the culture medium. The electrophysiological changes observed after acute exposure of different cell types to Irvalec suggest that the compound is able to modify the plasma membrane in such a way that it becomes permeable to ions (e.g Ca^2+^ and Mn^2+^) even when the cell is not pulsed (since the holding current was changed from ∼10 pA to ∼10 nA).

These results are in agreement with previous data reported by other authors for Irvalec and Kahalalide F in other cell systems [3]–[5], [7], [22]. In yeast systems, Irvalec treatment was related to the formation of plasma membrane invaginations and necrotic cell death [7]. The activity of Kahalalide F against the protozoan *Leishmania* spp was recently associated to the alteration of the plasma membrane of the parasite [22]. In mammalian tumor cells, Kahalalide F was reported to cause changes in the osmotic balance of the cell that would finally induce cytoplasmic swelling and oncotic cell death [3], [4]. These authors postulated that the mechanism of action of Kahalalide F was similar to that of other cytotoxic peptides which induce cell death through the formation of pores in the membrane and/or by changing the activity of existing channels [5], [23].

To further demonstrate the important role of membrane disorganization in the cytotoxicity of Irvalec, we preincubated the tumor cells with zinc salts, which have been demonstrated to have membrane-stabilizing properties [24]. We show here that Zn^2+^ exerted a significant protective effect against the cytotoxicity of Irvalec as it prevented both the morphological changes associated with the necrotic process and the rapid permeabilization of the plasma membrane. Also, at the electrophysiological level, Zn^2+^ attenuated the Irvalec-induced alterations. In fact, Zn^2+^ significantly increases membrane anisotropy, which is accompanied by an increase in order parameter in membrane preparations. Therefore, we can speculate that this effect of ZnCl_2_ on the fluidity of the lipid bilayer avoids the effects of Irvalec.

The role of the cell membrane as the main target of Irvalec was subsequently confirmed in A549-Irv cells, a cell line that shows specific resistance to Irvalec. When treated with effective, necrotic concentrations of Irvalec, the resistant cells were completely insensitive. In fact, drug treatment did not induce any rise in [Ca^2+^]_cyt_ or any changes in membrane conductance or in membrane permeability thus indicating that these cells might have acquired specific alterations in their cell membrane that rendered them resistant to drug treatment. In yeast models, resistance to Irvalec was associated to the deficiency of the sphingolipid fatty acyl 2-hydroxylase Scs7 (orthologue to human FA2H) indicating that the presence of fatty acid 2-hydroxylation should be important for the maintenance of a membrane conformation required for drug activity [25], [26]. However, A549-Irv cells did not present any alteration in the expression of FA2H (data not shown). Experiments are currently underway aimed to identify the cell membrane alterations related to Irvalec resistance.

Using fluorescent derivatives of Irvalec, along with the lipophilic membrane biomarker PA-DPH and two-photon time-resolved imaging, we have demonstrated that the compound rapidly interacts with the plasma membrane of tumor cells, causing lipid bilayer restructuration and somehow altering its fluidity and normal physicochemical properties. Of interest, the interaction of fluorescent derivatives of Irvalec was not observed in A549-irv cells. Interestingly, it was also demonstrated, by using FRET, that at cytotoxic concentrations Irvalec molecules are forming some kind of assemblies all throughout the plasma membrane where the individual molecules of the drug are close enough to each another (less than 50 Å) as to let us suggest that the compound self-organizes in the plasma membrane, forming supramolecular structures that could trigger the disruption of membrane integrity.

The idea of Irvalec forming a supramolecular organization in the membrane to exert its cytotoxic action is in agreement with its behaviour in the cytotoxicity experiments, where it would need first to reach a critical local concentration to self-organize and form the cytotoxic structures that finally produce the drastic lytic effect. Other explanation for this particular behavior could be the fact that eukaryotic cells have specific and efficient mechanisms to overcome and repair sporadic membrane insults coming from quite different sources. Tumor cells could be triggering these repair mechanisms in the presence of low concentrations of Irvalec, totally counteracting the cytotoxic action of the compound, while at higher concentrations of the drug, the repair mechanisms are overcome resulting in rapid cell death. Ca^2+^ plays an important role in this repair mechanism, mediating exocytosis of proximal lysosomal vesicles to clamp the membrane at the sites of damage. This process is thought to be mediated by a rapid increase of intracellular Ca^2+^, most likely due to activation of extracellular Ca^2+^ uptake by specific mechanisms. Supporting this idea, we found that Irvalec rapidly alters Ca^2+^ dynamics in treated cells, promoting a rise in cytosolic Ca^2+^ concentrations. This increase was mainly due to Ca^2+^ entry, indicating cell membrane permeabilization after drug treatment. In some cells (those that would finally die by necrosis) the Ca^2+^ increase was quickly followed by cell permeabilization in all the cells. These dramatic changes in Ca^2+^ permeability across the plasma membrane may contribute to explain its dose-dependent toxicity.

Most conventional anticancer agents need to enter cancer cells in order to exert their cytotoxic activity. On the other hand, cancer cells frequently become resistant to these agents as a consequence of increased expression of drug efflux pumps or drug-detoxifying enzymes or the appearance of defects in the cellular apoptotic machinery. Thus, the development of new classes of anticancer drugs that act at the cell surface and that are unaffected by common mechanisms of chemoresistance would be a major advance in cancer treatment. One of the major caveats that should encounter molecules acting at the cell membrane level is non-specificity towards tumor cells. In these sense, results from xenografted tumors in animal models and phase I clinical data demonstrated that Irvalec presents antitumor activity without inducing major adverse effects [1], [2]. Whether this is due to specific characteristics of the cell membrane of tumor cells or to subtle effects at non-cytotoxic concentrations that could drive to some more specific events in tumor cells is not known and studies are currently conducted in our laboratory to elucidate these matters.

In conclusion, our results strongly suggest that Irvalec rapidly and irreversibly targets the plasma membrane of tumor cells, altering its normal architecture and function. After reaching a critical, effective concentration, the compound appears to self-organize and give rise to highly cytotoxic molecular structures that trigger the lytic process characteristic of necrotic cell death.

## Supporting Information

Figure S1 **Chemical structure of Irvalec.**
(TIF)

Figure S2 **Effects of Irvalec on lysosomes.** A549 cells were pre-treated with both lysotracker, to label the lysosomes, and AlexaFluor 488-conjugated beta subunit of cholera toxin, to label the plasma membrane, and then treated with Irvalec (10 µM). Representative images are shown.
(TIF)

Figure S3 **Representation of Mn^2+^ permeability records in A549 cells after treatment with 0.5 µM Irvalec.** The graph corresponds to the mean ± SEM values of [Ca^2+^]cyt in all cells (n = 11) for two different experiments.
(TIF)

Figure S4 **Analysis of P-glycoprotein expression in A549-Irv cells.** (A) Detection of P-glycoprotein protein expression by immunofluorescence using a specific antibody against this efflux pump (B) The functional activity of P-gp in A549 cells was analysed using the calcein-AM method. Briefly, the fluorescent calcein-AM compound is a Pgp substrate that accumulates inside cells when they do not express the efflux pump (green fluorescence). In P-gp expressing cells, calcein-AM is rapidly effluxed and thus, cells did not show the green fluorescence. The figure shows representative images of calcein-AM accumulation in A549-Irv cells. The LoVo/Dox cells were used as a control of a P-glycoprotein positive cell line.
(TIF)

Figure S5 **Chemical structures of fluorescent Irvalec derivatives.** (**A**) Irvalec-Oregon Green (**B**) Irvalec-AlexaFluor 555.
(TIF)

Figure S6 **Localization of Irvalec in the plasma membrane of A549-Irv cells.** For these experiments, A549-Irv cells were grown on LabTek chambered slides and treated with 4.6 µM of a mix of 0.1 µM Irv-OG488 and non-labeled Irvalec ; cells were analyzed by two-photon time-resolved fluorescence microscopy (A) Representative 2P-FLIM image of a A549-Irv cell that did not stain with Irv-OG488 (B) Representative 2P-FLIM image of Irv-OG488 bound to the plasma membrane of a resistant cell. This cell represents a minority population (<5%) of resistant cells in which there was some interaction of Irvalec with the cell membrane.
(TIF)

Table S1 **Cytotoxic activity of a panel of anticancer drugs in A549 cells resistant to Irvalec (A549-Irv).** A549 and A549-Irv cell lines were treated with increasing concentrations of all compounds and the cytotoxic effect was determined by the MTT method after 72 h. Table shows mean IC_50_ values expressed in µM and the Relative Resistance Index (IR) of A549-Irv with regard to A549.
(DOC)

Video S1 **Induction of necrosis by Irvalec.** HeLa cells were treated with 10 µM Irvalec and examined by real time phase contrast video-microscopy.
(AVI)

Video S2 **Irvalec induces the formation of giant vesicles and necrosis.** A549 cells were stained with AlexaFluor 488-conjugated β-subunit of the cholera toxin to fluorescently label the plasma membrane and subsequently treated with 10 µM Irvalec.
(AVI)

Video S3 **Irvalec induces a rapid plasma membrane permeabilization.** Representative images of A549 cells exposed to 10 µM Irvalec for 2 min. In the presence of propidium iodide, unpermeabilized cells show intact nuclei while permeabilized cells show PI stained nuclei.
(AVI)

Video S4 **Irvalec induces a rapid plasma membrane permeabilization.** A549 cells were pre-loaded with calcein AM and then treated with 10 µM Irvalec. The intracellular fluorescence rapidly vanished from permeabilized cells upon Irvalec treatment.
(AVI)

Video S5 **Protective effects of Zinc against Irvalec cytotoxicity in tumor cells.** A549 cells were treated with 5 µM Irvalec alone or in combination with 10 mM ZnCl_2_, in the presence of propidium iodide. Cell permeabilization was followed by fluorescence microscopy upon PI nuclear staining.
(AVI)
