# Supplementary figures and images for: Irvalec Inserts into the Plasma Membrane Causing Rapid Loss of Integrity and Necrotic Cell Death in Tumor Cells
Source: PLoS One. 2011 Apr 27;6(4):e19042. doi: 10.1371/journal.pone.0019042 (PMC3083409; doi:10.1371/journal.pone.0019042)

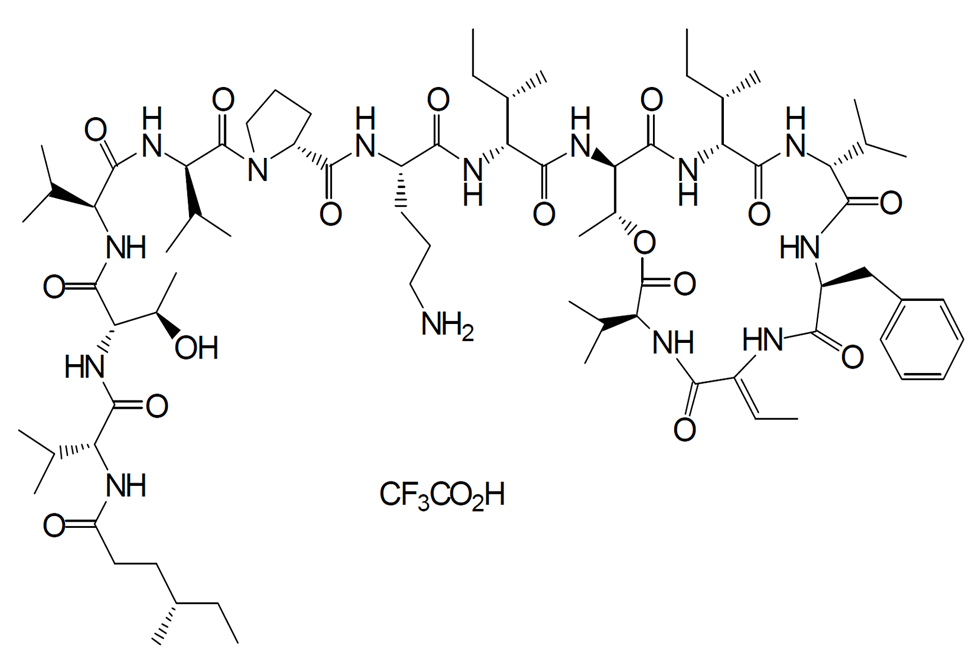

Supplement: Figure S1 — Chemical structure of Irvalec. (TIF) [file pone.0019042.s001.tif]

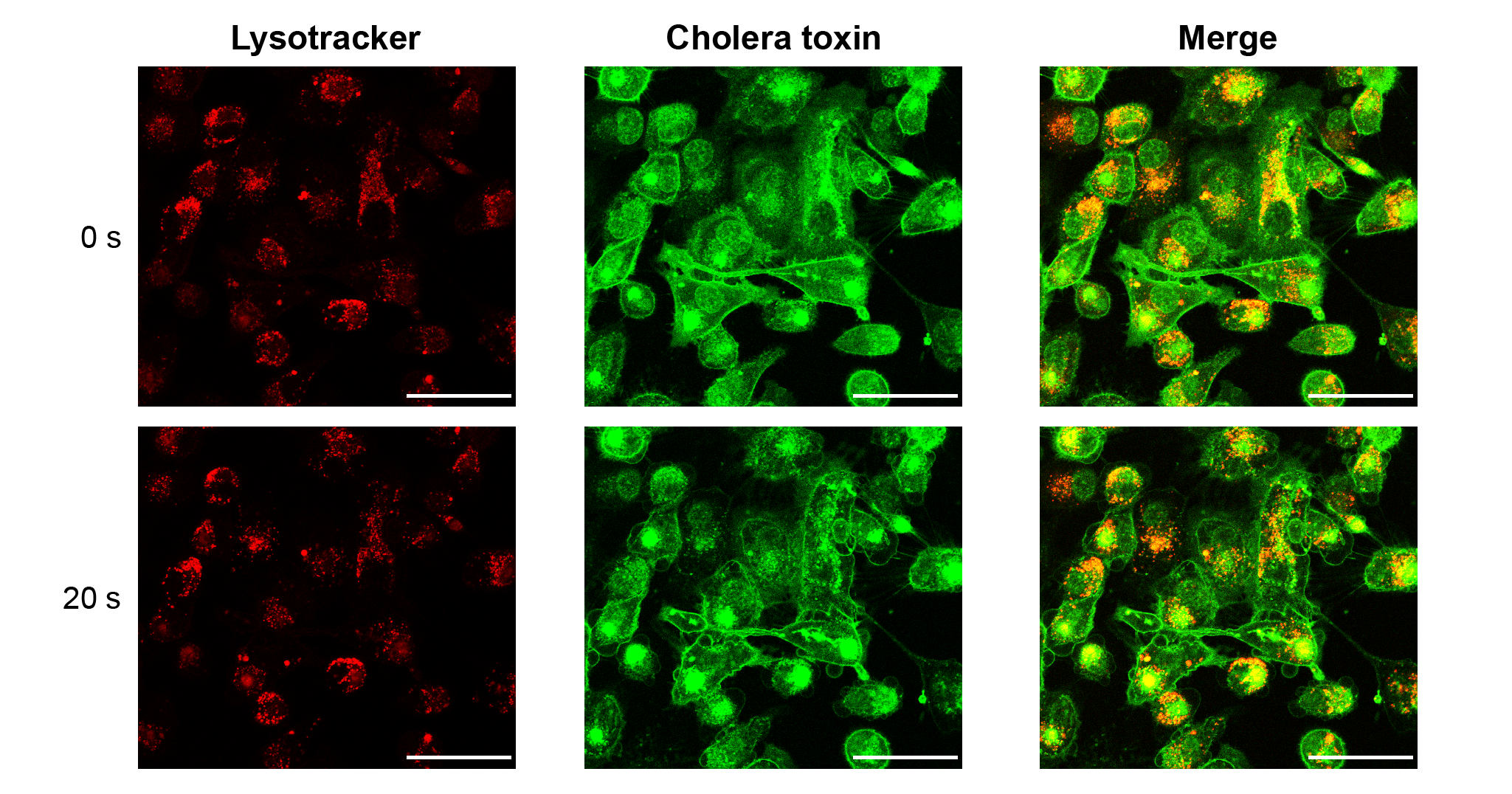

Supplement: Figure S2 — Effects of Irvalec on lysosomes. A549 cells were pre-treated with both lysotracker, to label the lysosomes, and AlexaFluor 488-conjugated beta subunit of cholera toxin, to label the plasma membrane, and then treated with Irvalec (10 µM). Representative images are shown. (TIF) [file pone.0019042.s002.tif]

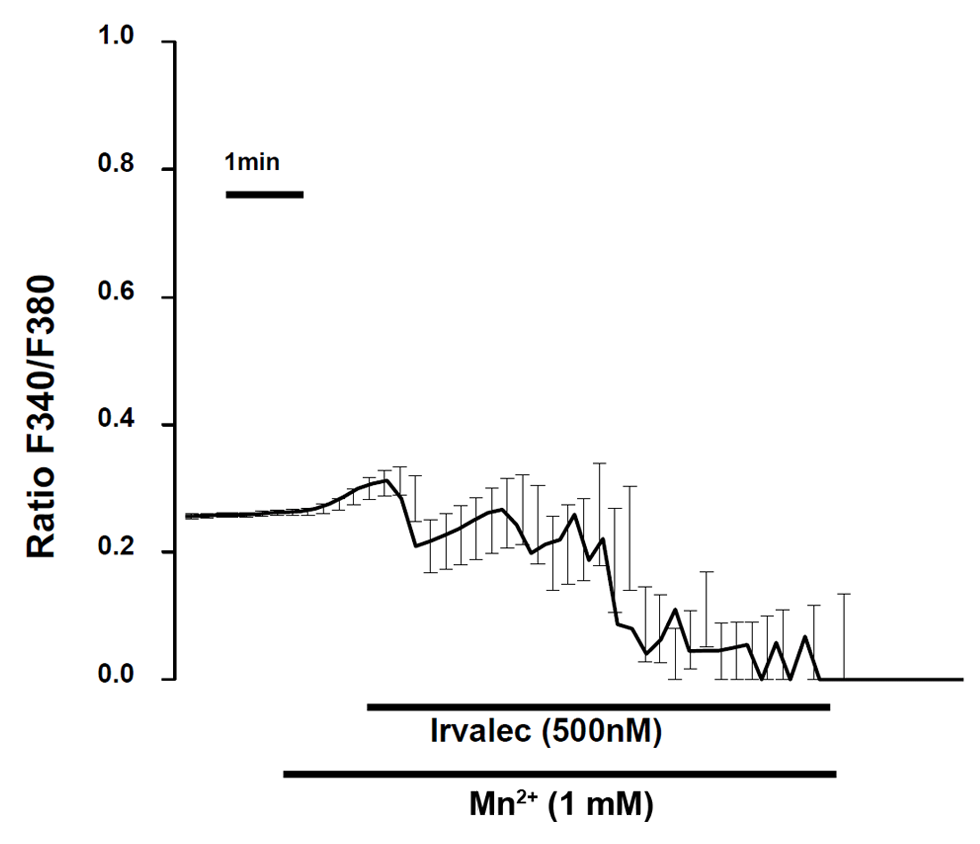

Supplement: Figure S3 — Representation of Mn2+ permeability records in A549 cells after treatment with 0.5 µM Irvalec. The graph corresponds to the mean ± SEM values of [Ca2+]cyt in all cells (n = 11) for two different experiments. (TIF) [file pone.0019042.s003.tif]

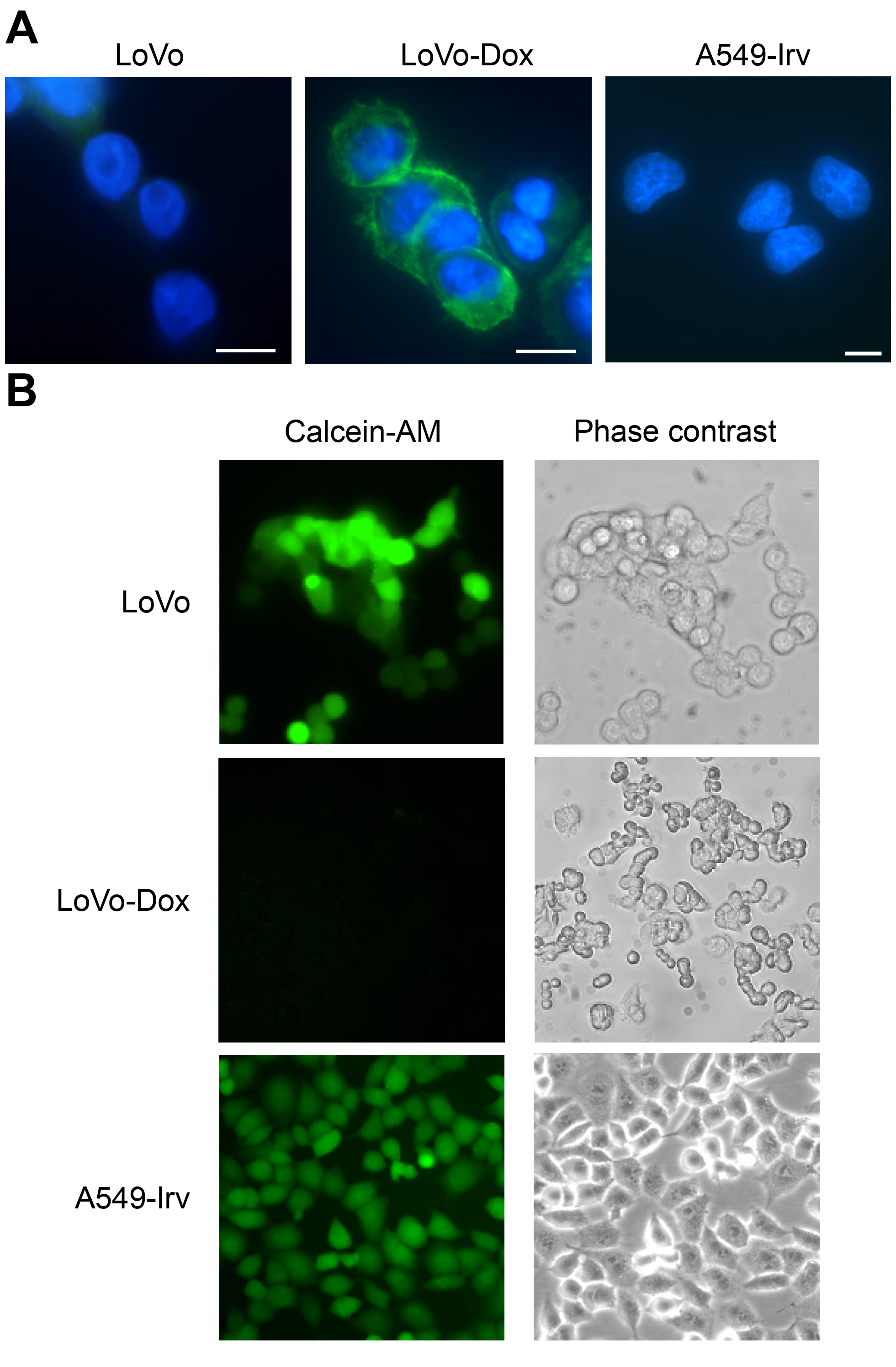

Supplement: Figure S4 — Analysis of P-glycoprotein expression in A549-Irv cells. (A) Detection of P-glycoprotein protein expression by immunofluorescence using a specific antibody against this efflux pump (B) The functional activity of P-gp in A549 cells was analysed using the calcein-AM method. Briefly, the fluorescent calcein-AM compound is a Pgp substrate that accumulates inside cells when they do not express the efflux pump (green fluorescence). In P-gp expressing cells, calcein-AM is rapidly effluxed and thus, cells did not show the green fluorescence. The figure shows representative images of calcein-AM accumulation in A549-Irv cells. The LoVo/Dox cells were used as a control of a P-glycoprotein positive cell line. (TIF) [file pone.0019042.s004.tif]

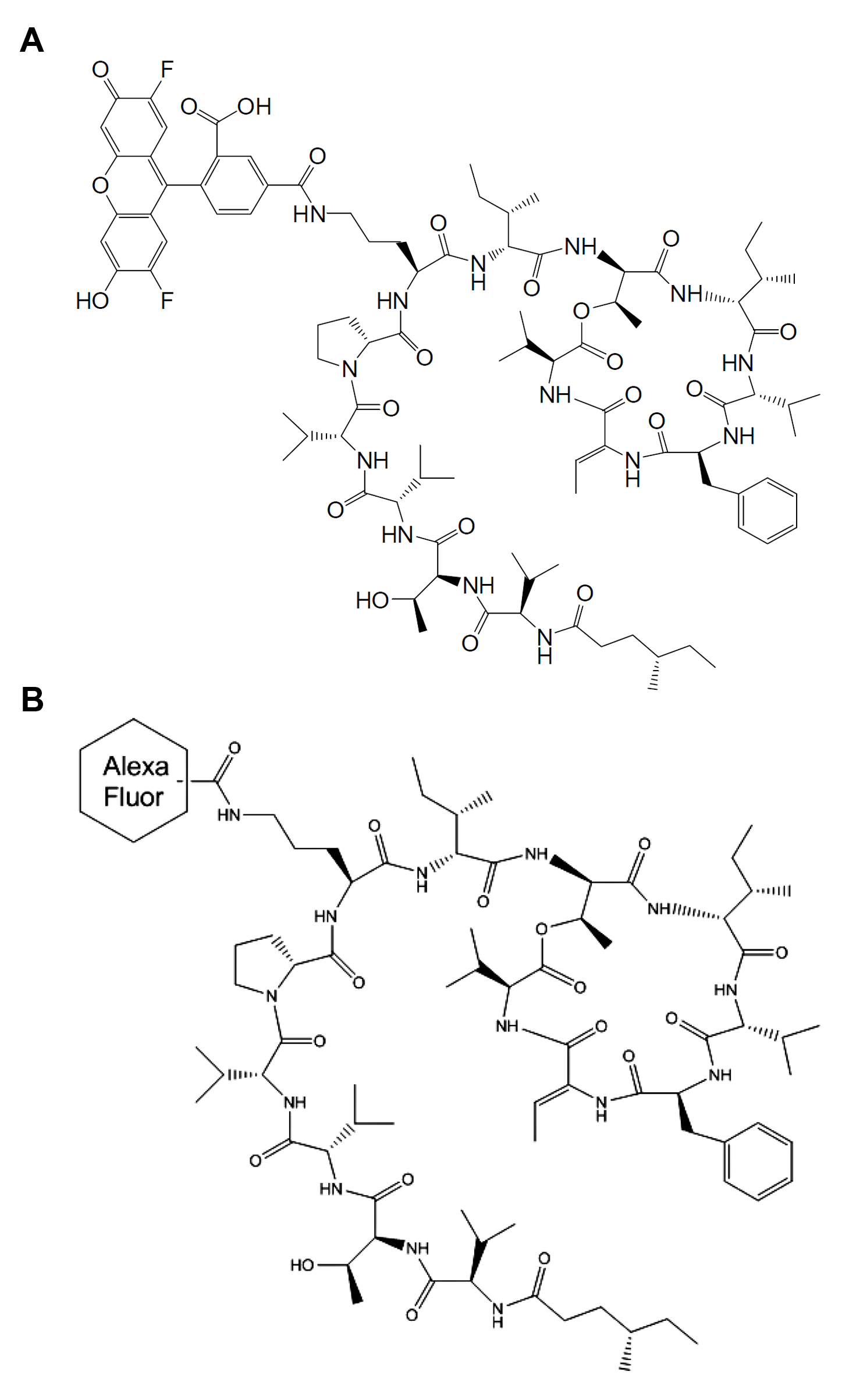

Supplement: Figure S5 — Chemical structures of fluorescent Irvalec derivatives. (A) Irvalec-Oregon Green (B) Irvalec-AlexaFluor 555. (TIF) [file pone.0019042.s005.tif]

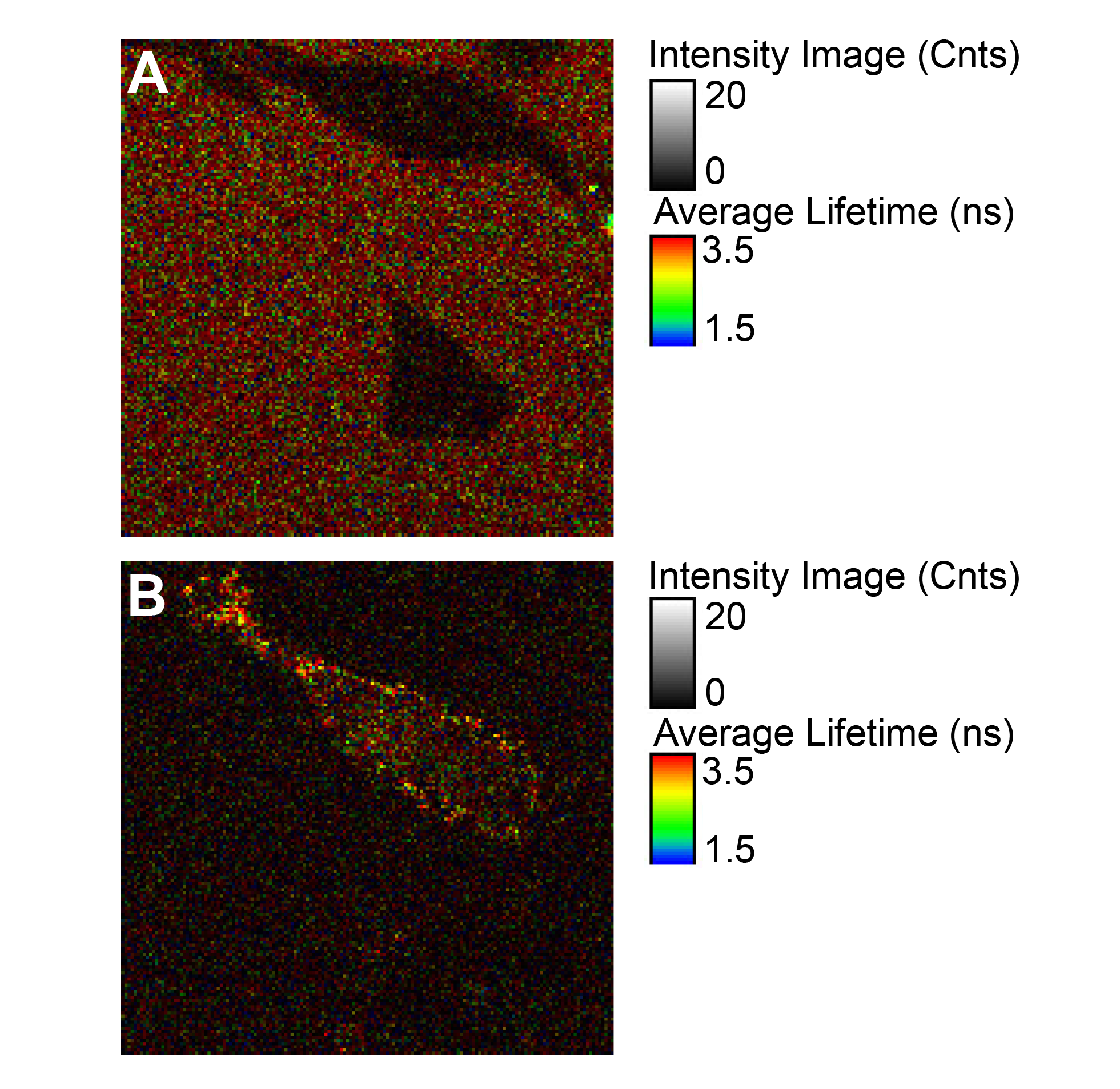

Supplement: Figure S6 — Localization of Irvalec in the plasma membrane of A549-Irv cells. For these experiments, A549-Irv cells were grown on LabTek chambered slides and treated with 4.6 µM of a mix of 0.1 µM Irv-OG488 and non-labeled Irvalec ; cells were analyzed by two-photon time-resolved fluorescence microscopy (A) Representative 2P-FLIM image of a A549-Irv cell that did not stain with Irv-OG488 (B) Representative 2P-FLIM image of Irv-OG488 bound to the plasma membrane of a resistant cell. This cell represents a minority population (<5%) of resistant cells in which there was some interaction of Irvalec with the cell membrane. (TIF) [file pone.0019042.s006.tif]
