# Supplementary material for: Irvalec Inserts into the Plasma Membrane Causing Rapid Loss of Integrity and Necrotic Cell Death in Tumor Cells
Source: PLoS One. 2011 Apr 27;6(4):e19042. doi: 10.1371/journal.pone.0019042 (PMC3083409; doi:10.1371/journal.pone.0019042)
Supplement: Table S1 — Cytotoxic activity of a panel of anticancer drugs in A549 cells resistant to Irvalec (A549-Irv). A549 and A549-Irv cell lines were treated with increasing concentrations of all compounds and the cytotoxic effect was determined by the MTT method after 72 h. Table shows mean IC50 values expressed in µM and the Relative Resistance Index (IR) of A549-Irv with regard to A549. (DOC) [file pone.0019042.s007.doc]

**Table S1. Cytotoxic activity of a panel of anticancer drugs in A549 cells resistant to Irvalec (A549-Irv)**

|  | **A549** | **A549-Irv** | **IR** |
| --- | --- | --- | --- |
| **Mitomycin C** | 1.15 | 1.05 | -1.1 |
| **Cisplatin** | 65.3 | 74 | -1.1 |
| **5-Fluorouracil** | 15.9 | 8.63 | -1.8 |
| **Gemcitabine** | 0.0006 | 0.0006 | -1.0 |
| **Etoposide** | 1.24 | 0.78 | -1.6 |
| **Paclitaxel** | 0.019 | 0.004 | -4.8 |
| **Docetaxel** | 0.005 | 0.001 | -3.4 |
| **Epothilone B** | 0.0004 | 0.0005 | 1.3 |
| **Vinorelbine** | 0.29 | 0.076 | -3.9 |
| **Vinblastine** | 0.008 | 0.002 | -3.8 |
| **Plitidepsin** | 0.002 | 0.002 | 1 |

A549 and A549-Irv cell lines were treated with increasing concentrations of all compounds and the cytotoxic effect was determined by the MTT method after 72 h. Table shows mean IC_50_ values expressed in µM and the Relative Resistance Index (IR) of A549-Irv with regard to A549
